# Correction: Development of an indirect ELISA for the detection of venezuelan equine encephalitis virus specific antibodies in horses

**DOI:** 10.1371/journal.pone.0358506

**Published:** 2026-09-15

**Authors:** The PLOS One Staff

## Notice of Republication

This article was republished on August 18, 2026, to correct an error in the author list that was introduced during the typesetting process. The publisher apologizes for the errors. Please download this article again to view the correct version. The originally published, uncorrected article and the republished, corrected articles are provided here for reference.

## Supporting information

S1 File Originally published, uncorrected article. (PDF)

S2 File Republished, corrected article. (PDF)
