# Supplementary figures and images for: Development of an indirect ELISA for the detection of venezuelan equine encephalitis virus specific antibodies in horses
Source: PLoS One. 2026 Jun 10;21(6):e0338819. doi: 10.1371/journal.pone.0338819 (PMC13252768; doi:10.1371/journal.pone.0338819)

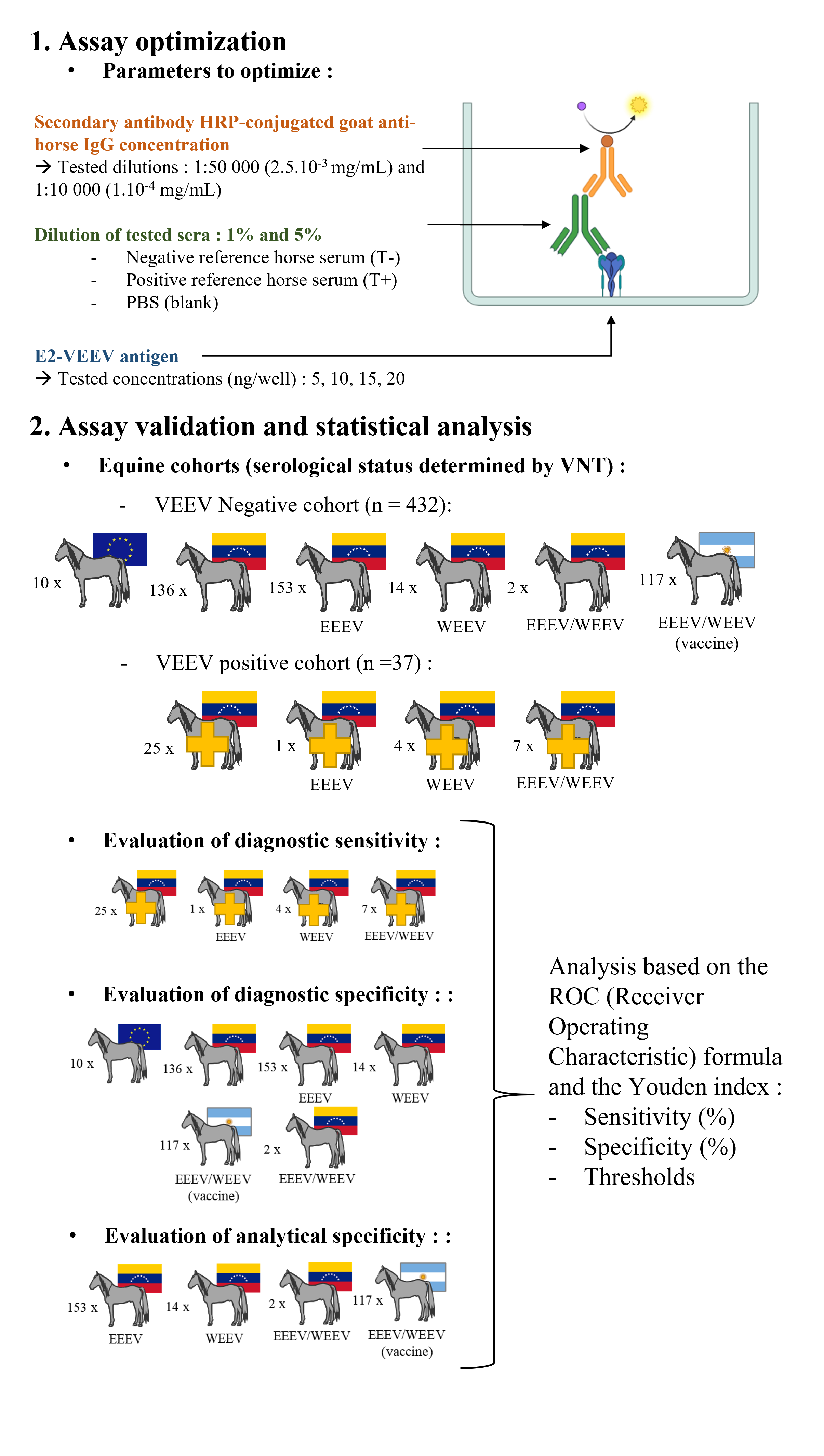

Supplement: S1 Fig — (TIF) [file pone.0338819.s001.tif]
