# Supplementary material for: Correction: Development of an indirect ELISA for the detection of venezuelan equine encephalitis virus specific antibodies in horses
Source: PLoS One. 2026 Sep 15;21(9):e0358506. doi: 10.1371/journal.pone.0358506 (PMC13577377; doi:10.1371/journal.pone.0358506)
Supplement: Supplementary file 1 [file pone.0358506.s001.pdf]

PLOS One

RESEARCH ARTICLE

Development of an indirect ELISA for the detection of venezuelan equine encephalitis virus specific antibodies in horses

Manon Chevrier[1], Laure Mathews-Martin[1], Teheipuaura Mariteragi-Helle[1], Marine Dumarest[1], Yasmine Boudjadi[1], Emmanuel Bréard[1], Mathilde Turpaud[1], Thomas Deshayes[1], María Aldana Vissani[2,3,4], Rommel Lenin Vinueza[5], Roberto Coello Peralta[6], Jessica Vanhomwegen[7], Stephan Zientara[1], Cécile Beck[1], Sandra Martin-Latil[1], Philippe Desprès[8], Gaëlle Gonzalez[1], Camille V. Migné[1]*

1 Anses, INRAE, Ecole Nationale Vétérinaire d'Alfort, UMR 1161 Virologie, Laboratoire de Santé Animale, Maisons-Alfort, France, **2** Instituto de Virología, CICVyA, Instituto Nacional de Tecnología Agropecuaria (INTA), Buenos Aires, Argentina, **3** Facultad de Ciencias Agrarias y Veterinarias, Instituto de Investigación en Veterinaria, Universidad del Salvador, Buenos Aires, Argentina, **4** Instituto de Virología e Innovaciones Tecnológicas, UEDD, CONICET, Buenos Aires, Argentina, **5** Escuela de Medicina Veterinaria, Universidad San Francisco de Quito, Quito, Ecuador, **6** Facultad de Medicina Veterinaria y Zootecnia, Universidad de Guayaquil, Guayaquil, Ecuador, **7** Institut Pasteur, Université Paris Cité, Unité Environnement et Risques Infectieux, Paris, France, **8** Processus Infectieux en Milieu Insulaire Tropical (PIMIT), Université de La Réunion, INSERM U 1187, CNRS9192, IRD249, Plateforme Technologique CYROI, Sainte-Clotilde, La Réunion, France

* camille.migne@anses.fr

Abstract

Venezuelan equine encephalitis virus (VEEV) is a re-emerging zoonotic pathogen to which equines are most susceptible mammals hosts with a high mortality rate raging from19 to 83%. The virus, transmitted between vertebrates and mosquitos, causes unpredictable sporadic epizootic outbreaks in equids and humans across Central and South America. Although VEEV is not currently circulating in Europe the combination of global change, increasing international trade and the presence of potentially competent vectors (mosquitoes of the genera *Aedes* and *Culex*), raises a non-negligible risk of its introduction. In response, the European Commission mandated the European Union Reference Laboratory (EURL) for equine diseases to establish an easy-to-implement and fast serological method for detecting VEEV infection in horses. The study aimed to develop a sensitive and specific *in-house* indirect enzyme-linked immunosorbent assay (ELISA) based on the E2 glycoprotein of VEEV. The assay was evaluated using 469 samples from non-infected horses, naturally infected horses (with VEEV and/or eastern equine encephalitis virus (EEEV) and/or western equine encephalitis virus (WEEV)), and horses vaccinated against EEEV/WEEV. The ELISA demonstrated robust diagnostic performance with a sensitivity of 97.3% and a specificity and 93.8%. This assay is therefore an easy-to-implement diagnostic method, offering an alternative to virus neutralization. It is suitable for sero-epidemiological studies to accurately determine the distribution

Check for updates

OPEN ACCESS

Citation: Chevrier M, Mathews-Martin L, Mariteragi-Helle T, Dumarest M, Boudjadi Y, Bréard E, et al. (2026) Development of an indirect ELISA for the detection of venezuelan equine encephalitis virus specific antibodies in horses. PLoS One 21(6): e0338819. https://doi.org/10.1371/journal.pone.0338819

Editor: Shih-Chao Lin, National Taiwan Ocean University, TAIWAN

Received: July 24, 2025

Accepted: November 27, 2025

Published: June 10, 2026

Copyright: © 2026 Chevrier et al. This is an open access article distributed under the terms of the Creative Commons Attribution License, which permits unrestricted use, distribution, and reproduction in any medium, provided the original author and source are credited.

Data availability statement: All relevant data are within the paper and its Supporting Information files.

Funding: This work was supported by the European Commission – European Union Reference Laboratory for Equine Diseases (to

MC, LMM, TMH, MD, YB, SZ, CB, SML, GG, CVM).

Competing interests: The authors have declared that no competing interests exist.

of VEEV in the America and to monitor potential introduction of the virus in new area, includingEurope.

Introduction

Venezuelan equine encephalitis virus (VEEV), a member of the family *Togaviridae*, genus *Alphavirus*,is the causative agent of Venezuelan equine encephalitis, a mosquito-borne disease affecting all equine species (horses, mules, donkeys) as well as humans [1].

VEEV is a reemerging pathogen endemic to Central and South America with recent outbreaks reported in Mexico, Venezuela and Colombia. The virus was first identified in 1935 following equine outbreaks in Venezuela, Colombia, and Trinidad [2] and was subsequently isolated in humans in 1950 [2] during an outbreak in Colombia. Since its discovery, VEEV has caused sporadic epidemic outbreaks that can extend over wide geographic area and persist for several months or even years [2]. The virus is highly pathogenic in both equines and humans, with mortality in equids ranging from 19% to 83% and neurological complications reported in 4% to 14% of human cases [3]. Notably, past outbreaks involving epizootic strains have affected as many as 75,000 individuals during a single epidemic [2].

The VEEV genome is a single-stranded, positive-sense, spherical, enveloped RNA virus, 65−70 nm in diameter, with an icosahedral capsid [4]. The genome contains two open reading frames (ORFs) that are prone to genetic recombination. The first ORF encodes 4 non-structural proteins(nsP-1, nsP-2, nsP-3, and nsP-4), while the second encodes 5 structural proteins(C, E1, E2, E3, and 6K) [5]. Most alphaviruses are maintained in a transmission cycle alternating between vertebrate hosts which serve as reservoirs and arthropod vectors, typically mosquitoes [6,7]. VEE complex alphaviruses are grouped into six antigenic subtypes based primarily on E2 protein epitopes: IAB, IC, ID, IE, IF, and II-IV. The virus exhibits two epidemiological forms. Enzootic strains (subtypes ID, IE IF, and II–VI) circulate between rodents and *Culex* mosquitoes. Epizootic strains (usually subtypes IAB and IC), involve an amplification cycle in which humans and horses serve as amplifying hosts with mosquitoes of the genera *Aedes* and *Psorophora* acting as vectors [5]. During infection, equines and humans develop sufficient viremia to transmit the virus to a naïve mosquito during its blood meal with viremia typically lasting 3–4 days and occasionally persisting up to 3 weeks [8].

VEEV has been designated a biothreat agent in the Americas by the Centers for Disease Control and Prevention [9]. Biothreat agents are classified into 3 categories based on their mode of dissemination, transmission, mortality rates, and potential public health impact. VEEV is categorized as a B-level biothreat agent because aerosolized particles are highly infectious and can cause higher mortality than natural infection via mosquito bites [10]. Exposure to as few as 10–100 infectious aerosolized particles can induce symptomatic disease ranging from mild illness to neurological symptoms, in nearly all humans [11]. Furthermore, no approved vaccine or therapeutics are currently available for human use, highlighting its potential as a

bioterrorism threat. Consistently, the National Institute of Allergy and Infectious Diseases (NIAID) also classifies VEEV as a category B pathogen due to the biosafety risk it poses.

VEEV is currently absent in Europe. However, global warming, which alters mosquito ecology [12], the intensification of international trade including legal and illegal animal movements, transports of infected animals or vectors, international events,…) and the presence of potentially competent mosquito vectors such as *Aedes* spp. and *Culex* spp. raise the risk of emergence of VEEV in Europe [8,13]. To manage the introduction of emerging diseases, the European Commission has classified such diseases under the Animal Health Law of April 2021 [14]. In this framework, Category A includes exotic diseases requiring immediate eradication upon introduction, Category B includes diseases subject to compulsory eradication, Category C comprises diseases targeted for voluntary eradication, Category D includes diseases subject to trade restrictions to prevent spread within the EU and Category E comprises diseases requiring compulsory surveillance. VEEV has been classified as categories D and E, meaning it must be declared and monitored in Europe, and trade between Member States must be restricted, in the event of its introduction.

The European Commission mandated the European Union Reference Laboratory (EURL) for equine diseases with developing serological detection tools for VEEV in accordance with the WOAH manual (World Organization for Animal Health) including Indirect Haemagglutination Test (IHA), Virus Neutralization Test (VNT), and ELISA. However, handling live VEEV requires a biosafety level 3 laboratory (BSL3) due to its zoonotic potential, airborne transmissible, and the absence of approved treatment or vaccine. Among available assays, the VNT remains the gold standard because of its high specificity and sensitivity but it is a time-consuming, labor-intensive and requires highly trained personnel operating under biosafety level 3 laboratories (BSL3) conditions. To facilitate safer and more accessible testing at the national level, we aimed to develop an indirect ELISA that can be implemented in BSL2 laboratories. For this purpose, a recombinant VEEV E2 glycoprotein, a highly immunogenic protein that elicits neutralizing antibodies was used as antigen [8].

An indirect ELISA was subsequently developed to detect VEEV-infected animals. The assay was validated and its performance was evaluated using samples previously characterized with the gold standard VNT, allowing assessment of sensitivity, specificity and overall reliability of the test.

Materials and methods

The flowchart of the analysis performed is available in the supplementary data S1 Fig.

Equine sera

The origin and the serological status of the 469 serum samples analysed in this study are summarized in Table 1. Samples collected from South America (Ecuador and Argentina) and Europe were classified into three categories: those from

Table 1. Origins, serological status and infection methods of sera from horses in the study.

Number of sera	Origins	Serological status (VNT)	Animal status
25	Ecuador	VEEV	Naturally infected
1		VEEV; EEEV	
4		VEEV; WEEV	
7		VEEV; EEEV; WEEV	
2		EEEV/WEEV	
153		EEEV	
14		WEEV	
136		Negative	Naïve
117	Argentina	EEEV; WEEV	Vaccination
10	Europe	Negative	Naïve

https://doi.org/10.1371/journal.pone.0338819.t001

individuals naturally infected by VEEV, EEEV and/or WEEV, those vaccinated against EEEV and WEEV or those from non-infected individuals.

Sera from horses naturally infected with VEEV and/or EEEV and/or WEEV (206 samples) as well as sera from non-infected horses (136 samples) from Ecuador were kindly provided by Dr Rommel Lenin Vinueza Sierra, Universidad San Francisco de Quito (Laboratorio de Entomología Médica y Medicina Tropical (LEMMT)).

In addition, 20 VEEV-WEEV-EEEV seronegative horses were vaccinated with two doses of Zoetis' Fluvac Innovator 4 vaccine (USA) administered 15 days apart and sampled on the day of the first injection (day 0) and on days 7, 15, 21, 28 and 36. Hundred seventeen samples were provided by Dr Aldana Vissani (Instituto Nacional de Tecnología Agropecuaria, Buenos Aires).

Furthermore, 10 VEEV, EEEV and WEEV negative European sera from the biobank of the EURL for equine diseases were included in this study.

The serological status for each sample had been determined prior to this work by VNT for eastern, western and Venezuelan according to the standard operating procedure developed by the EURL for equine diseases [15,16].

VEEV E2 glycoprotein

A synthetic gene coding for the soluble form of VEEV E2 glycoprotein (sE2) from strain IAB strain (Genbank access n° KC344483.2) was cloned into the Drosophila expression vector pMT/BiP/HisA in which the SNAP-tag sequence (Covalys BioSciences AG, France) had been inserted (15). The synthetic gene was delivered by Genecust (Boynes, France). In the construct, the VEEV sE2 protein was fused in frame with the N-terminus of SNAP-tag protein. The SNAP-tagged sE2 protein was purified from the S2 cell supernatant as it has been previously described [15]. Briefly, the sample was injected onto a 5 mL TALON affinity column (GE Healthcare) and, after washing the column, the protein was eluted with an imidazole gradient up to a concentration of 200 mM. Next, the collected fractions (containing the protein) were pooled, concentrated, and injected at 1 mL/min onto a HiLoad 16/60 Superdex 75 PG column (GE Healthcare) equilibrated in buffer [20 mM Tris pH 8.0; 200 mM NaCl]. The quality of the purified protein fractions was verified by migrating the fractions in a 4–15% polyacrylamide gradient gel (Mini-PROTEAN® TGX Stain-Free™ precast gel, Bio-Rad). These proteins were visualized using a ChemiDoc™ MP imager (Bio-Rad). The fractions containing the purified proteins of interest were collected, aliquoted, and stored at −80°C. Protein quantification used the Bradford method with the Bio-Rad Protein Assay kit (Bio-Rad). Production batches yielded between 7 and 30 mg of glycoprotein E2. A qualitative Quality Check (QC) of the purified proteins was performed by measuring the absorbance between a 240–340 nm range using a UV-VIS spectrophotometer (Jasco) in order to calculate the 260/280 ratio and the aggregation index to ensure >95% protein purity and absence of nucleic acid contamination. Protein integrity and absence of protein contaminants was verified by confirming the molecular mass by MALDI-TOF/TOF mass spectrometry (Bruker).

Throughout this manuscript, the protein is designated as E2-VEEV antigen.

Development of the E2 VEEV Indirect ELISA

The optimal concentration of the E2 antigen was determined by checkerboard titration of the E2-VEEV antigen using VEEV-positive sera (from Ecuador) and negative or vaccinated horse sera. The dilution of the secondary antibody, an HRP-conjugated goat anti-horse IgG (Euromedex, France) was also optimized. Based on these results, the E2-VEEV antigen was diluted in PBS (Phosphate buffered saline, Thermo Fisher Scientific, USA) and coated at 15 ng/well. Sera were diluted to 1% and the secondary antibody was used at 1:10 000 dilution (1.10^{-4} mg/mL) in PBS (Thermo Fisher Scientific, USA)-Tween 20 0.05% (Sigma-Aldrich)-milk 5% (Thermo Fisher Scientific) – BSA (Bovine Serum Albumin) 1% (Sigma) for both.

Plates were coated with the full-length glycoprotein E2 at concentration ranging from 5 to 20 ng per well and ELISA was performed using two sera dilutions (1:100 and 1:20)), and three secondary antibody dilutions (1:100 000, 1: 50 000 and 1:10 000) (Fig 1).

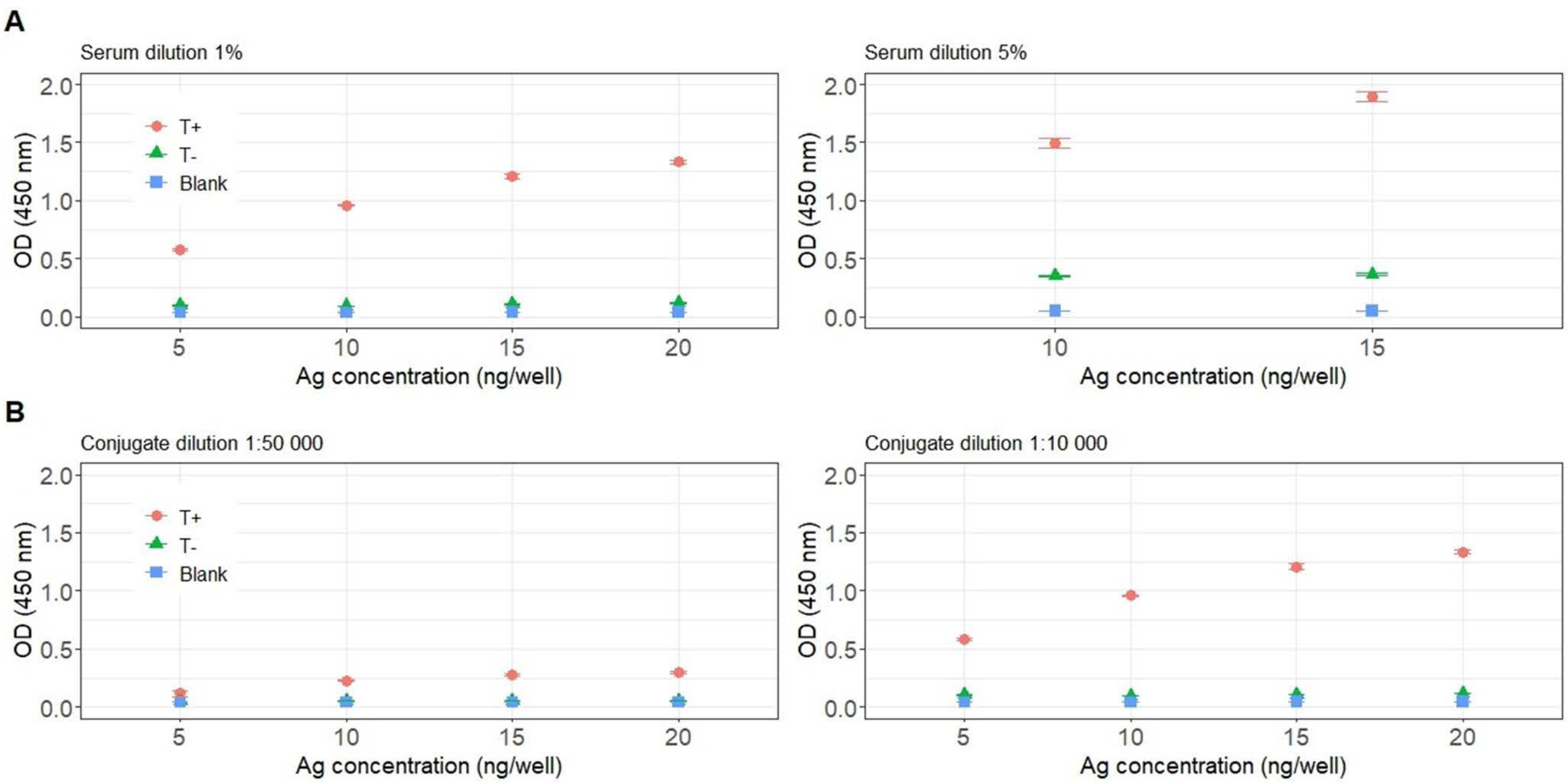

Fig 1. Identification of optimal parameters for an Indirect ELISA using VEEV glycoprotein E2. The graphs present absorbance values at 450 nm (y-axis) as a function of antigen concentration per well (ng/well) (x-axis), under fixed serum dilution and secondary antibody concentration. In both panels **(A)** and **(B)**, PBS was used as blank (blue square), while negative (T-, green triangles) and positive (T+, red dots) sera were included correspond negative and as VNT control sample. **(A)** Two serum dilutions (1% and 5%) were tested. For serum diluted at 1%, four coating concentrations were evaluated; for sera diluted at 5%, two coating concentration were assessed. The secondary antibody was diluted 1:10 000, **(B)** Two conjugate dilutions were tested across four coating concentrations with serum diluted at 1%.

https://doi.org/10.1371/journal.pone.0338819.g001

The optimized conditions were determined by identifying the highest optical density (OD) difference between positive and negative samples while maintaining the lowest OD for negative samples compared to the blank.

After overnight incubation at 4°C, and a wash with 140 µL / well of PBS, each well was blocked with 150 µL/well of PBS-Tween 20 0.05%-milk (Thermo Fisher Scientific, USA) 5%- BSA 1% solution (Sigma-Aldrich, USA) for 30 min at room temperature. Plates were then washed three times with 250 µL/well of PBS containing 0.1% Tween 20, 50 µL of each serum sample diluted at 1:100 were added per well and incubated for 2h at 4°C. Following three washes with PBS-Tween 20 0.1% (250 µL/well) 50 µL/well of HRP-conjugated goat anti-horse IgG, diluted at 1:10 000, was added and incubated for 1h at room temperature. After 3 additional washes, 75 µL of tetramethylbenzidine reagent (TMB) (IDVet, France) was added per well and plates were incubated at room temperature in the dark for 10 minutes. The reaction was stopped by adding 75 µL/well of 0.5 M H_2SO_4 (IDVet, France). Optical density was measured at 450 nm using the Multiskan FC plate reader (Thermo Fischer Scientific, USA). ELISA results were recorded with SkanIt RE 4.1 software and expressed as raw optical density values. Each sample was analyzed once, reflecting a typical diagnostic ELISA setting, with each run including a negative and a positive serum in duplicate to validate the assay.

Statistical analysis

The threshold of the test was calculated using R software (version 4.3.3) based on the ROC (Receiver Operating Characteristic) formula and the Youden index in the Epi package computing sensitivity, specificity, tested thresholds, and AUC (Area Under the Curve) [17]. The 37 positive sera from Ecuador and 432 VEEV-negative sera (originated from Argentina (117/432), Ecuador (305/432) and Europe (10/432)) were processed to obtain the cutoff. The selected threshold value

represents the optimal compromise between sensitivity and specificity. ELISA sensitivity was estimated using 37 positive sera. Reported seroprevalence of VEEV in Latin America varies widely, ranging from 36% in equines in Costa Rica, 14.1% in humans in Argentina (Chaco province), and 18–75% in equines in Mexico [18–20]. In Ecuador, our previous VNT-based study revealed a positivity rate of 10.8% among 342 equids. According to *Bujang et al.*, at least 34 positive samples are required for diagnostic tool validation within a cohort of this size, consistent with our dataset [21]. Specificity was assessed using 432 negative sera from Ecuador, Argentina and Europe, providing a robust evaluation precise and reliable assessment of the ability of our ELISA to correctly identify true negatives [21]. Cross-reactivity with EEEV and WEEV was assessed on Argentinian vaccinated sera and EEEV and WEEV naturally infected horses from Ecuador.

Ethics statement

The animal studies in Ecuador were approved by Agencia de Regulación y Control de la Bioseguridad y Cuarentena paraGalápagos (ABG), Puerto Ayora, Ecuador. The studies were conducted in accordance with the local legislation and institutional requirements. Written informed consent was obtained from the owners for the participation of their animals in this study.

Vaccinated horse sera from Argentina belong to the Biobank of the EURL for equine diseases and were kindly provided by Dr Aldana Vissani, head of the Laboratory of Equine Virology, National Institute of Agrotechnology (INTA), Buenos Aires, Argentina.

Results

The datasets used and/or analysed during the current study are available from the corresponding author on reasonable request. All relevant data are within the paper and its Supporting Information files (S2 File.).

Optimization of ELISA assay

Based on checkerboard titration assays, the ELISA have been performed using E2-VEEV antigen diluted in PBS (Phosphate buffered saline, Thermo Fisher Scientific, USA) and coated at 15 ng/well, sera diluted at 1% and secondary antibody at 1:10 000 (1.10^{-4} mg/mL) (Fig 1). These conditions were selected considering the preciousness of sera and the satisfactory results obtained with the concentration of 15 ng/well of E2-VEEV antigen, which were similar to those achieved with a glycoprotein concentration of 20 ng/well.

ELISA results

A total of 469 horse sera were tested to assess the performance of the ELISA test. Hundred seventeen Argentinian horses sera vaccinated against EEEV/WEEV as well as 9 out of 10 European horse sera had OD value below 0.3. Almost all of the 305 VEEV-naïve horse sera from Ecuador had an OD of less than 0.3. Twenty-six of these sera had an OD greater than 0.3 (Fig 2). Seven of them were VEEV/EEEV/WEEV naïve and the other were EEEV VNT-positive. The ELISA results show also that 36 out of 37 VEEV VNT-positive sera (mono- or co-infected with EEEV and/or WEEV) had an $OD \geq 0.3$. The cut-off of the test was calculated at 0.305, using ROC formula.

Analytical specificity. To evaluate the analytical specificity of the assay, 286 horse sera from WEEV/EEEV vaccinated horses ($n = 117$) and naturally EEEV and/or WEEV infected horses (EEEV: $n = 153$, WEEV: $n = 14$, EEEV/WEEV: $n = 2$) were tested to evaluate the analytical specificity of the assay. Serial sampling sera collected from vaccinated (against WEEV/EEEV) Argentinian horses ($n = 117$ samples) had ODs < 0.3 (Fig 3). These ODs values were similar to the ones obtained for negative sera from Ecuador (Fig 2). Sera collected from naturally infected horses by EEEV and WEEV showed a wider range of OD values ($0 < OD < 0.3$ for the majority). Nineteen out of the 153 sera collected from naturally EEEV infected horses were found positive by the ELISA assay (Figs 2 and 3). Horses with WEEV and EEEV/WEEV

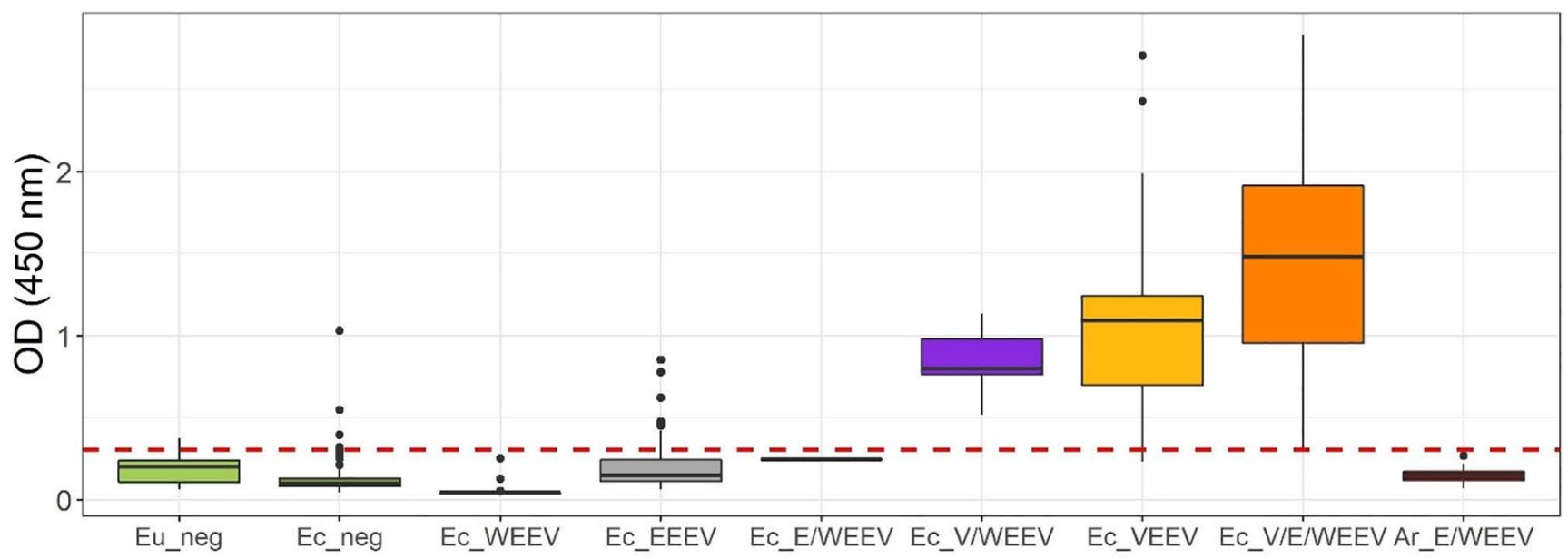

Fig 2. Absorbance values at 450 nm obtained with the E2 VEEV indirect ELISA (y-axis) according to the serological status of the equids (x-axis) (A total of 469 horses from Ecuador, Argentina and Europe were included, distributed among VNT- defined groups: naturally VEEV infected (n = 25), co-infected VEEV/EEEV (n = 1) or VEEV/WEEV (n = 4) or VEEV/EEEV/WEEV (n = 7) or EEEV/WEEV (n = 2), mono-infected with WEEV (n = 14) or EEEV (n = 153) and non-infected (n = 136 from Ecuador, n = 10 from Europe). The dashed red horizontal line indicates the ELISA cut-off (OD = 0.305). Eu: European horses, Ec: Ecuadorian horses, Ar: Argentinian horses, neg: negative status.

https://doi.org/10.1371/journal.pone.0338819.g002

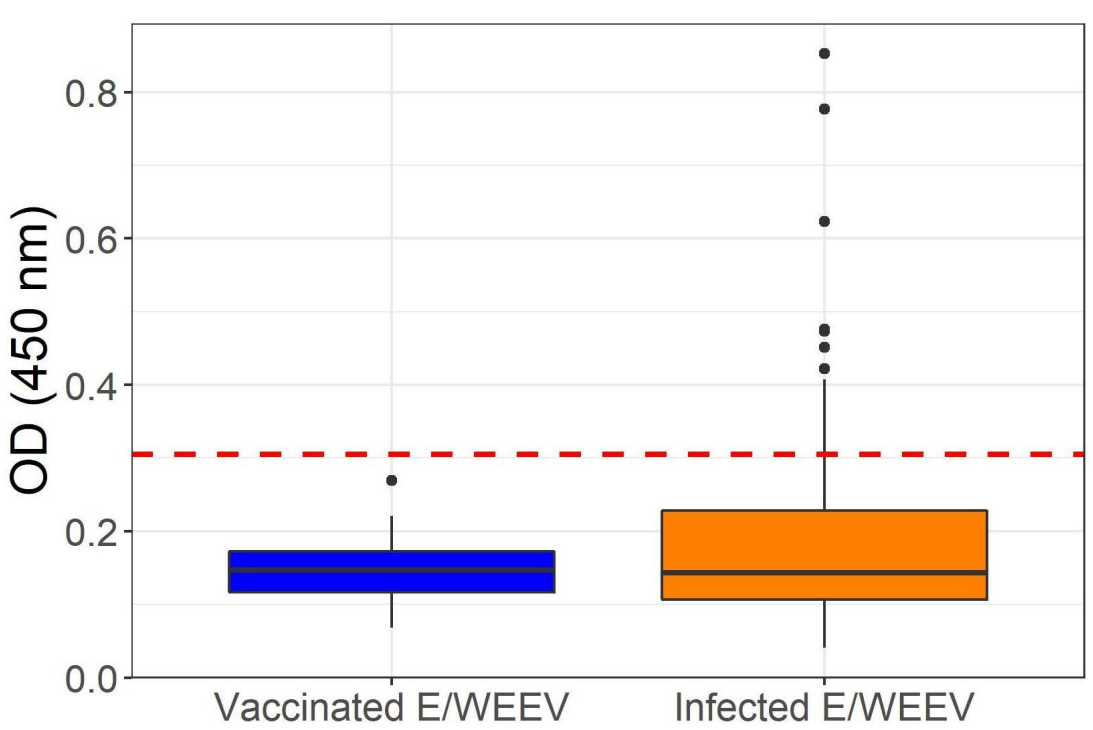

Fig 3. Absorbance values at 450 nm obtained with the E2 VEEV indirect ELISA (y-axis) according to the serological status of the equids (x-axis). Results are shown for 286 sera including Ecuadorian horses naturally infected with EEEV (n = 153), WEEV (n = 14), or co-infected with EEEV/WEEV (n = 2) and Argentinian horses vaccinated against EEEV and WEEV (n = 117).

https://doi.org/10.1371/journal.pone.0338819.g003

serological status were found negative. The false positive rate is low and estimated at 4.05% for this population including vaccinated and naturally infected horses.

Diagnostic sensitivity and specificity. To determine the diagnostic performance of the assay, thirty-six out of the thirty-seven naturally infected VEEV horse sera were detected ELISA-positive (97.3%) (Table 2). Similarly, 405 out of 432 VEEV-negative sera confirmed by VNT were identified ELISA-negative (93.8%). As a whole, 441 out of 469 horse sera (94%) had concomitant VNT and ELISA results. The specificity of this ELISA is satisfactory, estimated at 93.8% (95% CI [91.0%−95.8%]) and the sensitivity assessed at 97.3% (95% CI [85.8%−100%]).

Discussion

This study, conducted by the EU-RL for arthropod-borne equine encephalitis viruses within the European mandate on equine diseases, aimed to develop an indirect *in-house* ELISA test for the detection of IgG antibodies against VEEV using the highly immunogenic E2 glycoprotein as antigen [22]. At present, VEEV only circulates on the American continent and causes unpredictable outbreaks in horses and humans [2]. As arboviruses are constantly expanding their geographical distribution, due to globalization, the intensification of international trade, global warming, etc. the risk of VEEV

Table 2. VEEV ELISA and VNT results from 469 horses.

VEEV ELISA		VNT Results		
		**Positive**	**Negative**	**Total**
Results	**Positive**	36	27	63
	**Negative**	1	405	406
	**Total**	37	432	469

https://doi.org/10.1371/journal.pone.0338819.t002

introduction in Europe is increasing. To anticipate its emergence in Europe, it was necessary to develop a safe serological diagnostic tool usable in BSL2 by all Member States. The optimized condition includes coating plates with 15 ng of E2 glycoprotein per well, dilution of screening sera at 1:100, and use of conjugate diluted at 1:10 000. A higher concentration of coating antigen (20 ng/well) did not improve the OD at 450 nm. A lower concentration of coating antigen (10 ng/well) decreased the analytical sensitivity of the test. The positivity threshold, set at 0.305, was determined from 469 horse sera using the ROC formula. While analyzing the data, we noted that some sera had OD values close to the positivity threshold (0.305), which raises questions about the need to include an interval of doubtful values in the ELISA. In the event of a doubtful result, a second blood sample from the suspected horse is requested at least two weeks after the first sampling in order to confirm whether an infection occurred.

We successfully developed an ELISA tool offering 97.3% sensitivity and 93.8% specificity showing improved performance compared to another ELISA-VEEV test developed under similar conditions (serum dilution and conjugate concentration) but using the whole VEEV particle from the inactivated vaccine strain TC83 as antigen [18]. Indeed, León and colleagues (Leon *et al.*, 2020) analyzed 243 Costa Rican horse sera with their ELISA based on VNT assay which achieved a sensitivity of 88% and a specificity of 86%. Wang *et al.,* developed in 2005 a competition ELISA test for VEEV allowing the differentiation between enzootic (IE and ID) and epizootic (IAB and IC) strains of the virus. Despite its advantages, it is not available as a cost-effective, easy to implement diagnostic assay. The test was designed with whole viral particle as antigen (from four different serotypes: IAB, IC, IE and ID) and the use of monoclonal antibodies specific to epizootic or enzootic strains which allowed differentiation between the strains [23].

The sensitivity was determined by testing 37 VEEV-positive sera from Ecuadorian horses. Thirty-six sera had an OD ≥ 0.305. Only 1 VEEV positive serum confirmed by VNT was detected negative using the indirect in-house VEEV ELISA test. We delineate a VEEV VNT titer ≥ 20 to report a VNT positive result. This VEEV-positive serum has a VNT-VEEV titer of 10 w may explain the false negative result obtained by ELISA. All positive sera in our study originated from Ecuador, as obtaining equine field samples from other countries was not possible which may limit the generalizability of our findings. However, the literature indicates that VEEV strains are antigenically close, with little to no variation in antibody responses. Thus, despite the limited geographic origin of samples, our results remain relevant for broader VEEV contexts.

The specificity of the test was evaluated testing 305 Ecuadorian sera sampled from non-infected or naturally infected horses (EEEV and/or WEEV) as well as 117 sera from EEEV-WEEV vaccinated Argentinian horses and 10 sera from European horses. The serological status of these sera (Fig 2 and 3) was determined performing VNT for VEEV, EEEV and WEEV. The majority of VEEV-naive sera (405 out of 432) had an OD < 0.305 and 27 sera have ODs > 0.305. Among these 27 horses, 26 originated from Ecuador. It is important to note that VNT is a method that detects specific neutralizing antibodies against viruses contained in serum, whereas an ELISA test may detect all antibodies undetectable by VNT. Our VNT results demonstrated that VEEV, WEEV, and EEEV co-circulate in Venezuela. Moreover, the only available data in the literature, regarding the persistence of VEEV-neutralizing antibodies were obtained from horses vaccinated with the TC-83 vaccine, which contains an attenuated VEEV strain. Studies have shown that antibodies persist for at least 18 months in all horses tested in the study by *Vanderwagen LC et al* [24]. In another study, nearly 50% of the cohort no

longer exhibited neutralizing antibodies 14 months post-vaccination [25]. It cannot be ruled out that horses with a serological status positive for EEEV may have been previously infected with VEEV, and that neutralizing antibodies are no longer detectable by VNT. This difference may explain the variations in results between VNT and ELISA (ELISA serum-positive but VNT-negative and vice versa). The owners did not report any history of disease for these horses, in addition, no prior information was provided on Ecuadorian horses' serological history, randomly sampled from the field. Horses may have been infected by circulating neglected-arboviruses [26] and especially other alphaviruses in Ecuador (CHIKV, EEEV and WEEV), which may cross-react with the test. Serological cross-reactions between EEEV and VEEV have been previously reported in a study conducted on horses in Trinidad, where both viruses co-circulate. The authors favored the hypothesis of cross-reactivity over that of co-infection for 1.4% of their cohort of 506 horses [27]. Even though VEEV and CHIKV are phylogenetically distant, rare serological cross-reactions have been demonstrated in patients between VEEV and CHIKV [28,29]. Finally, the last serum found positive by ELISA (OD = 0.377) was from a European horse. As VEEV is a regulated disease and classified as categories D and E in Europe according to the Animal Health Law, the serological status will be confirmed by the EU-RL by carrying out a VNT for VEEV, EEEV and WEEV, in case of positive ELISA result found in a Member State.

Conclusion

Overall, the VEEV indirect ELISA based on E2 glycoprotein offers satisfactory results in terms of sensitivity and specificity. This technique offers an easy to implement and fast alternative to virus neutralization test performed in biosafety level 3 laboratories. In the future, it could be interesting to adapt this protocol to diagnose VEEV infection in a wide variety of species, such as human or other animal species that could be used as sentinels for surveillance, and to extend it to efficiently detect eastern and western equine encephalitis virus infection.

Finally, this tool meets the requirements of the European Commission. It is easy to implement, accessible to European Union Member State and provides additional security for laboratory staff. Moreover, they offer genuinely innovative solutions for keeping Europe prepared in case of introduction of Venezuelan equine encephalitis virus on the continent, notably through international events.

Supporting information

S1 Fig. Schematic overview of the process employed for optimizing and validating the ELISA assay.
(TIF)

S2 File. Raw data.
(XLSX)

Author contributions

Conceptualization: Gaëlle Gonzalez, Camille Victoire Migné.

Formal analysis: Manon Chevrier, Laure Mathews-Martin.

Funding acquisition: Stephan Zientara, Gaëlle Gonzalez.

Investigation: Manon Chevrier, Teheipuaura Mariteragi-Helle, Marine Dumarest, Yasmine Boudjadi.

Methodology: Emmanuel Bréard, Mathilde Turpaud, Sandra Martin-Latil, Gaëlle Gonzalez, Camille Victoire Migné.

Project administration: Gaëlle Gonzalez, Camille Victoire Migné.

Resources: María Aldana Vissani, Rommel Lenin Vinueza, Roberto Coello Peralta, Jessica Vanhomwegen, Philippe Desprès, Gaëlle Gonzalez, Camille Victoire Migné.

Supervision: Camille Victoire Migné.

Validation: Gaëlle Gonzalez, Camille Victoire Migné.

Visualization: Gaëlle Gonzalez, Camille Victoire Migné.

Writing – original draft: Manon Chevrier.

Writing – review & editing: Laure Mathews-Martin, Emmanuel Bréard, Mathilde Turpaud, Thomas Deshayes, María Aldana Vissani, Rommel Lenin Vinueza, Roberto Coello Peralta, Jessica Vanhomwegen, Stephan Zientara, Cécile Beck, Sandra Martin-Latil, Philippe Desprès, Gaëlle Gonzalez, Camille Victoire Migné.

References

1. Guzmán-Terán C, Calderón-Rangel A, Rodriguez-Morales A, Mattar S. Venezuelan equine encephalitis virus: the problem is not over for tropical America. Ann Clin Microbiol Antimicrob. 2020;19(1):19. https://doi.org/10.1186/s12941-020-00360-4 PMID: 32429942
2. Crosby B, Crespo ME. Venezuelan equine encephalitis. StatPearls. Treasure Island (FL): StatPearls Publishing. 2024.
3. Zacks MA, Paessler S. Encephalitic alphaviruses. Vet Microbiol. 2010;140(3–4):281–6. https://doi.org/10.1016/j.vetmic.2009.08.023
4. Jose J, Snyder JE, Kuhn RJ. A structural and functional perspective of alphavirus replication and assembly. Future Microbiol. 2009;4(7):837–56. https://doi.org/10.2217/fmb.09.59 PMID: 19722838
5. Weaver SC, Winegar R, Manger ID, Forrester NL. Alphaviruses: Population genetics and determinants of emergence. Antiviral Research. 2012;94(3):242–57. https://doi.org/10.1016/j.antiviral.2012.04.002
6. Chen R. ICTV Virus Taxonomy Profile: Togaviridae. J Gen Virol. 2018;99(6):761–2. https://doi.org/10.1099/jgv.0.001072
7. Go YY, Balasuriya UBR, Lee C-K. Zoonotic encephalitides caused by arboviruses: transmission and epidemiology of alphaviruses and flaviviruses. Clin Exp Vaccine Res. 2014;3(1):58–77. https://doi.org/10.7774/cevr.2014.3.1.58 PMID: 24427764
8. Dumas I. Risque d'introduction des encéphalites équines "exotiques" (encéphalite japonaise, encéphalite vénézuélienne, encéphalites américaines de l'Est et de l'Ouest) en France. 2013.
9. CDC. Bioterrorism Agents/Diseases (by category). Emergency Preparedness & Response. https://emergency.cdc.gov/agent/agentlist-category.asp
10. Franz DR. Clinical recognition and management of patients exposed to biological warfare agents. JAMA. 1997;278(5):399–411. https://doi.org/10.1001/jama.278.5.399
11. Rusnak JM. Approach to strain selection and the propagation of viral stocks for Venezuelan equine encephalitis virus vaccine efficacy testing under the animal rule. Viruses. 2019;11(9):807. https://doi.org/10.3390/v11090807
12. El-Sayed A, Kamel M. Climatic changes and their role in emergence and re-emergence of diseases. Environ Sci Pollut Res Int. 2020;27(18):22336–52. https://doi.org/10.1007/s11356-020-08896-w PMID: 32347486
13. Weaver SC, Reisen WK. Present and future arboviral threats. Antiviral Res. 2010;85(2):328–45. https://doi.org/10.1016/j.antiviral.2009.10.008
14. Occitanie D. 01 - La nouvelle catégorisation des maladies animales et ses impacts. 2024. https://draaf.occitanie.agriculture.gouv.fr/01-la-nouvelle-categorisation-des-maladies-animales-et-ses-impacts-a5791.html
15. Beck C. A high-performance multiplex immunoassay for serodiagnosis of flavivirus-associated neurological diseases in horses. BioMed Res Int. 2015;2015:678084. https://doi.org/10.1155/2015/678084
16. European Union Reference Laboratory for Equine Diseases. EURL. 2024. https://eurl-equinediseases.anses.fr/
17. López-Ratón M, Rodríguez-Álvarez MX, Cadarso-Suárez C, Gude-Sampedro F. Optimal Cutpoints: An R Package for Selecting Optimal Cutpoints in Diagnostic Tests. J Stat Softw. 2014;61:1–36. https://doi.org/10.18637/jss.v061.i08
18. León B, Käsbohrer A, Hutter SE, Baldi M, Firth CL, Romero-Zúñiga JJ, et al. National Seroprevalence and Risk Factors for Eastern Equine Encephalitis and Venezuelan Equine Encephalitis in Costa Rica. J Equine Vet Sci. 2020;92:103140. https://doi.org/10.1016/j.jevs.2020.103140 PMID: 32797803
19. Pisano MB, Oria G, Beskow G, Aguilar J, Konigheim B, Cacace ML, et al. Venezuelan equine encephalitis viruses (VEEV) in Argentina: serological evidence of human infection. PLoS Negl Trop Dis. 2013;7(12):e2551. https://doi.org/10.1371/journal.pntd.0002551 PMID: 24349588
20. Adams AP. Venezuelan equine encephalitis virus activity in the Gulf Coast region of Mexico, 2003–2010. PLoS Negl Trop Dis. 2012;6(11):e1875. https://doi.org/10.1371/journal.pntd.0001875
21. Bujang MA, Adnan TH. Requirements for Minimum Sample Size for Sensitivity and Specificity Analysis. J Clin Diagn Res. 2016;10(10):YE01–6. https://doi.org/10.7860/JCDR/2016/18129.8744 PMID: 27891446
22. Weger-Lucarelli J, Aliota MT, Wlodarchak N, Kamlangdee A, Swanson R, Osorio JE. Dissecting the Role of E2 Protein Domains in Alphavirus Pathogenicity. J Virol. 2015;90(5):2418–33. https://doi.org/10.1128/JVI.02792-15 PMID: 26676771

23. Wang E, Paessler S, Aguilar PV, Smith DR, Coffey LL, Kang W, et al. A novel, rapid assay for detection and differentiation of serotype-specific antibodies to Venezuelan equine encephalitis complex alphaviruses. Am J Trop Med Hyg. 2005;72(6):805–10. https://doi.org/10.4269/ajtmh.2005.72.805 PMID: 15964967

24. Vanderwagen LC, Pearson JL, Franti CE, Tamm EL, Riemann HP, Behymer DE. A field study of persistence of antibodies in California horses vaccinated against western, eastern, and Venezuelan equine encephalomyelitis. Am J Vet Res. 1975;36(11):1567–71. https://doi.org/10.2460/ajvr.1975.36.11.1567 PMID: 1190598

25. Walton TE, Alvarez O Jr, Buckwalter RM, Johnson KM. Experimental infection of horses with an attenuated Venezuelan equine encephalomyelitis vaccine (strain TC-83). Infect Immun. 1972;5(5):750–6. https://doi.org/10.1128/iai.5.5.750-756.1972 PMID: 4637604

26. Rodriguez-Morales AJ, Bonilla-Aldana DK. Neglected arboviruses in Latin America. New advances in neglected tropical diseases. IntechOpen. 2022. https://doi.org/10.5772/intechopen.108940

27. Thompson NN, Auguste AJ, Coombs D, Blitvich BJ, Carrington CVF, da Rosa APT, et al. Serological evidence of flaviviruses and alphaviruses in livestock and wildlife in Trinidad. Vector Borne Zoonotic Dis. 2012;12(11):969–78. https://doi.org/10.1089/vbz.2012.0959 PMID: 22989182

28. McClain DJ, Pittman PR, Ramsburg HH, Nelson GO, Rossi CA, Mangiafico JA, et al. Immunologic interference from sequential administration of live attenuated alphavirus vaccines. J Infect Dis. 1998;177(3):634–41. https://doi.org/10.1086/514240 PMID: 9498442

29. Powers JM, Lyski ZL, Weber WC, Denton M, Streblow MM, Mayo AT, et al. Infection with chikungunya virus confers heterotypic cross-neutralizing antibodies and memory B-cells against other arthritogenic alphaviruses predominantly through the B domain of the E2 glycoprotein. PLoS Negl Trop Dis. 2023;17(3):e0011154. https://doi.org/10.1371/journal.pntd.0011154 PMID: 36913428
